# Variants in matrix metalloproteinase‐9 gene are associated with hemorrhagic transformation in acute ischemic stroke patients with atherothrombosis, small artery disease, and cardioembolic stroke

**DOI:** 10.1002/brb3.1294

**Published:** 2019-05-10

**Authors:** Xingyang Yi, Guo Sui, Qiang Zhou, Chun Wang, Jing Lin, Zhenxiao Chai, Ju Zhou

**Affiliations:** ^1^ Department of Neurology People’s Hospital of Deyang City Deyang China; ^2^ Nursing Department People’s Hospital of Deyang City Deyang China; ^3^ Department of Neurology The Third Affiliated Hospital of Wenzhou Medical University Wenzhou China

**Keywords:** generalized multifactor dimensionality reduction, genetic variation, hemorrhagic transformation, ischemic stroke, *MMP‐9* gene

## Abstract

**Objective:**

The potential effect of matrix metalloproteinase‐9 (*MMP‐9*) variants and these variants interactions on hemorrhagic transformation (HT) risk after ischemic stroke (IS) remain unclear. The aims of present study were to investigate the associations of six variants in *MMP‐9* with HT, and these variants interactions whether related to increased HT risk.

**Method:**

A total of 705 patients with IS who were admitted to the participating hospitals within 48 hr of symptom onset were consecutively enrolled between March 2014 and December 2016. HT was confirmed by brain computed tomography (CT) scan during 14 days from stroke onset. Six variants of *MMP‐9* gene were measured by mass spectrometry. Interactions of gene variant–gene variant were assessed through generalized multifactor dimensionality reduction method (GMDR).

**Results:**

HT occurred in 104 (14.8%) patients. There were no differences in genotypes for the six variants between patients with and without HT using univariate analysis (all *p* > 0.05). GMDR analysis revealed that there was a synergistic effect of gene variant–gene variant interactions between rs3918242 and rs3787268 in *MMP‐9* gene. Cox regression analysis showed that high‐risk interactions of rs3918242 and rs3787268 were associated with increased risk of HT after adjusting for covariates (hazard ratio: 2.08; 95% confidence interval: 1.34–7.85; *p = *0.016).

**Conclusion:**

Incidence of HT is common in acute IS in Chinese population. The mechanisms leading to HT are most likely multifactorial. Two‐loci interactions of rs3918242 and rs3787268 in *MMP‐9* gene may confer a higher risk for HT.

## 1 BACKGROUND

Hemorrhagic transformation (HT) is the most feared complication in patient with acute ischemic stroke (IS). HT occurs in as many as 10%–40% of patients with IS (Beslow et al., 2011; Terruso et al., 2009). HT can exacerbate brain ischemic injury by promoting glial cell and neuronal death, and is associated with increased mortality and poor stroke outcomes (Khatri, Wechsler, & Broderick, 2007; Park et al., 2012). However, the underlying mechanisms for HT are unclear. Thus, to clarify the complex pathophysiology and basic mechanisms of HT within the context of stroke are essential to better confirm IS patients in prevention and treatment of HT, and reduce its impact on IS patients.

Blood–brain barrier (BBB) destruction is one of the major causes in development of HT in IS (Jickling et al., 2014). HT is associated with increased BBB permeability (Bang et al., 2007; Lin et al., 2007). The mechanisms of BBB breakdown in HT are not fully understood, although proteolytic degradation of neurovascular matrix and oxygen free radical damage are very important (Jickling et al., 2014; Kelly et al., 2008). Matrix metalloproteinase‐9 (MMP‐9) protein expression may increase after ischemia, and it plays an important role in BBB destruction (Barr et al., 2010). A number of studies have shown that high plasma concentration of MMP‐9 in acute phase of IS may increase HT risk within ischemic core (Castellanos et al., 2007; Jha et al., 2014; del Zoppo et al., 2012). Use of MMP‐9 inhibitors can decrease the BBB destruction and reduce the incidence of HT in experimental animals (Lu et al., 2009, 2008).

*MMP‐9* single nucleotide polymorphisms (SNPs) encode and regulate the transcription of MMP‐9 protein, and are associated with plasma MMP‐9 levels (Blankenberg et al., 2003). It has been known *MMP‐9* SNPs are associated with carotid atherosclerosis and increased IS incidence (Lin et al., 2012; Nie, Wang, & Tang, 2014; Yuan et al., 2013). Although many studies have revealed that there is an association of plasma MMP‐9 levels with HT risk (Castellanos et al., 2007; Jha et al., 2014; del Zoppo et al., 2012), the effect of *MMP‐9* SNPs on HT susceptibility is not clear. Zhang, Cao, Xu, Li, and Xu (2015) reported that *MMP‐9* variants were associated with HT of IS in the Chinese population. However, one study in a Mediterranean population did not show the associations of *MMP‐9* SNPs with HT (Montaner et al., 2003). These inconsistent results revealed that the genetic etiologies for HT may be complex, and common limitation of these studies is that these trials only assessed single gene polymorphisms without consideration of interactions between gene variant–gene variant. Nowadays, it is emphasized that investigation of gene variant–gene variant interactions is necessary to elucidate genetic mechanisms for the complex diseases by the generalized multifactor dimensionality reduction method (GMDR) (Lou et al., 2007). To date, the effect of gene variant–gene variant interactions in *MMP‐9* gene on HT risk is unknown.

In this study, we hypothesized the interactions among gene variant–gene variant in *MMP‐9* gene may affect HT risk in acute IS. To test this hypothesis, we measured six variants in *MMP‐9* to assess these gene variant–gene variant interactions whether increased the risk of HT. This study was earnestly hoped to provide insight into the mechanism for HT and better prevention of HT.

## 2 MATERIALS AND METHODS

### 2.1 Study population

Between March 2014 and December 2016, we consecutively enrolled 705 acute IS patients who were admitted to People's Hospital of Deyang City and the Third Affiliated Hospital of Wenzhou Medical University within 48 hr of symptom onset, and were confirmed by magnetic resonance imaging (MRI). All patients were performed brain computed tomography (CT) scan at admission and at 10–14 days of admission. Additional CT scans were performed whenever symptom deteriorated. Brain CT angiography or magnetic resonance angiography, carotid duplex ultrasound, electrocardiogram (ECG) or 24‐hr Holter ECG, echocardiogram were assessed in all patients. The inclusion criteria were as follows: (a) age ≥40 years old; (b) etiologies of IS were atherothrombosis, cardioembolism and small artery disease on the basis of classification system (Han et al., 2007). Exclusion criteria include: (a) undetermined or other etiologies of IS; (b) prestroke use of antiplatelet drugs within 7 days of stroke onset; (c) usage of warfarin or low‐molecular weight heparin or heparin before 7 days of stroke and within 14 days of symptom onset; (d) thrombolytic therapy, or thrombectomy; (e) intracranial hemorrhage (ICH) in area of non‐infarct; (f) malignant paraproteinemias, platelet count <100 × 10^9^/L; (g) severe liver, renal disease. The overall response rate was approximately 95% (705/741) for IS patients. This study was reviewed and approved by ethics committees of participating hospitals. The informed consent was obtained from each participant before study enrollment.

All patients were treated according to the standard guideline (Kernan et al., 2014). For each patient, National Institutes of Health Stroke Scale (NIHSS) score was performed by a member of stroke team at admission, and subsequently once a day during period of hospitalization. The various risk factors were recorded, including age, sex, hypertension, diabetes mellitus, atrial fibrillation, and blood pressure at presentation. Fasting glucose, total plasma cholesterol, triglycerides, low‐density lipoprotein cholesterol were measured (Yi, Han, Zhou, Lin, & Liu, 2016).

### 2.2 HT assessment

All enrolled patients were performed CT scan and MRI scan at admission, and a follow‐up CT at 10–14 days from stroke onset. On the basis of ECASS (European Cooperative Acute Stroke Study) classification (Larrue & Von, 1997), HT was defined as any degree of hyperdensity within the infarction zone during 14 days from stroke onset by brain CT scan.

### 2.3 Selection of SNPs and genotyping for MMP‐9

Variants of* MMP‐9* have been shown to influence MMP‐9 transcription and increase IS risk (Lin et al., 2012; Nie et al., 2014; Yuan et al., 2013; Zhang et al., 1999). We selected six variants of *MMP‐9* from the NCBI database (http://www.ncbi.nlm.nih.gov/SNP), including rs3918242, rs3787268, rs1056628, rs17576, rs2664517, and rs2250889. The inclusion criteria were as follows: (a) the variants of *MMP‐9* had been investigated in previous studies (Lin et al., 2012; Montaner et al., 2003; Nie et al., 2014; Yuan et al., 2013; Zhang et al., 2015); (b) according to HapMap data for Asian population (Altshuler et al., 2010), the minor allele frequency of tag major haplotypes >0.05.

Blood sample (3 ml) from each patient was drawn from an arm vein. DNA was extracted from the peripheral leukocytes. The six variants of *MMP‐9* were measured by matrix‐assisted laser desorption and ionization time‐of‐flight mass spectrometry method, as our previously described (Yi, Liao, Fu, Zhang, & Wang, 2015).

### 2.4 Statistical analysis

According to the suggested sample requirement of gene–gene interaction (Wang & Zhao, 2003), we calculated the sample in this study. Incidence of HT was about 10%–40% in patients with acute IS (Beslow et al., 2011; Terruso et al., 2009). According to the incidence of HT, we speculated a sample of 700 could provide 90% power to detect 10% relative risk increment of HT in patients harboring the high‐risk interactions genotype, assuming 10% rate of HT in IS patients carrying the low‐risk interactions genotype, if a two‐sided type I error of 0.05.

Hardy–Weinberg equilibrium for each variant and genotype distributions of the six variants between patients with and without HT was analyzed by chi‐squaredtest. Discrete variables were compared by chi‐squaredtest and continuous variables were compared by Student's *t* test between patients with and without HT. Gene–gene interaction was analyzed by GMDR method (β version 0.7, www.healthsystem.virginia.edu/internet/addiction-genomics/Software), as previously described (Lou et al., 2007; Yi et al., 2015).

The incidence of HT between patients with and without high‐risk interactive genotype was compared by chi‐squared test. Survival function estimate of HT was calculated using Kaplan–Meier analyses between patients with and without high‐risk interactive genotype. The risk factors for HT conferred by high‐risk interactive variable was analyzed by Cox proportional‐hazards model to adjust confounding variables, which were significantly associated with HT (*p* < 0.05) on univariate analysis, and were reported as hazard ratio (HR) with 95% confidence interval (CI).

## 3 RESULTS

### 3.1 Incidence of HT

Among the 705 enrolled patients, HT occurred in 104 (14.8%) patients during the 14 days after stroke onset. Baseline characteristics in patients with and without HT are summarized in Table 1. Old age, high NIHSS score at admission, ischemic areas, and cardioembolism were associated with HT by univariate analyses (Table 1).

**Table 1:** Characteristics of study patients

Characteristics	HT (*n* = 104)	Non‐HT (*n* = 601)	*p* Value
Age (years)	70.9 ± 13.2	68.0 ± 15.3	0.042
Men (*n*, %)	59 (56.7)	336 (55.9)	0.948
Hypertension (*n*, %)	83 (79.8)	468 (77.9)	0.675
Diabetes mellitus (*n*, %)	33 (31.7)	193 (32.1)	0.998
Atrial fibrillation (*n*, %)	16 (15.4)	63 (10.5)	0.159
Hyperlipidemia (*n*, %)	57 (54.8)	332 (55.2)	0.996
Systolic blood pressure (mm Hg)	154.7 ± 16.2	151.8 ± 18.9	0.098
Diastolic blood pressure (mm Hg)	90.2 ± 12.6	88.9 ± 16.8	0.393
Glucose (mM)	7.1 ± 2.4	7.2 ± 2.9	0.702
Onset to admission time (h)	29.8 ± 15.9	30.6 ± 18.6	0.678
NIHSS score at admission	11.2 ± 3.7	9.7 ± 3.6	<0.001
Ischemic areas (cm^2^)	4.6 ± 1.2	3.8 ± 1.5	<0.001
Stroke subtype (*n*, %)			
Atherothrombosis	50 (48.1)	358 (59.6)	<0.001
Small artery disease	23 (22.1)	152 (25.3)	
Cardioembolism	31 (29.8)	91 (15.1)	
In‐hospital treatment (*n*, %)			
Antihypertensive drugs	85 (81.7)	482 (80.2)	0.712
Hypoglycemic drugs	35 (33.7)	205 (34.1)	0.999
Statins	100 (96.2)	585 (97.3)	0.508
Aspirin	63 (60.6)	381 (63.4)	0.557
Aspirin plus clopidogrel	30 (28.8)	182 (30.3)	0.836

Abbreviation

HT, hemorrhagic transformation; NIHSS, National Institutes of Health Stroke Scale.

### 3.2 Genotype distributions in patients with and without HT

The distributions of the six variants for *MMP‐9* were in Hardy‐Weinberg equilibrium (*p* > 0.05). The genotype distributions of the six variants did not differ between patients with and without HT using single‐locus analytical method (*p* > 0.05 for each variant individually, Table 2).

**Table 2:** Genotype comparison between patients with and without HT (%)

		HT (*n* = 104)		Non‐HT (*n* = 601)	*P*‐value	
rs1056628						
AA	68 (65.4)		410 (68.7)			0.772
AC	28 (26.9)		162 (26.8)			
CC	8 (7.7)		29 (4.5)			
rs3918242						
CC	80 (76.9)		424 (70.5)			0.402
CT	19 (18.3)		148 (24.6)			
TT	5 (4.8)		29 (4.8)			
rs2664517						
CC	104 (100.0)		601 (100.0)			
rs17576						
AA	11 (10.6)		69 (11.5)			0.528
AG	37 (35.6)		236 (39.3)			
GG	56 (53.8)		296 (49.3)			
Rs3787268						
AA	43 (41.3)		240 (39.9)			0.251
AG	22 (21.2)		173 (28.8)			
GG	39 (37.5)		188 (31.3)			
rs2250889						
CC	62 (59.6)		333 (55.4)			0.987
CG	30 (28.8)		195 (32.4)			
GG	12 (11.5)		73 (12.1)			

Abbreviation

HT, hemorrhagic transformation.

### 3.3 Gene variant–gene variant interactions and HT

Then, we investigated the association between the high‐order interaction for the six variants and HT using the GMDR analysis. After adjusting covariates, the best interaction model for HT was rs3918242 and rs3787268, which cross‐validation consistency was scored 10/10, and the sign test was 9/10 (*p* = 0.019, Table 3). For each variant, one‐locus model was computed, and the significance of interaction was confirmed using permutation test (*p* = 0.031), indicating that interactions between two variants, rs3918242 and rs3787268, can synergistically contribute to higher risk for HT.

**Table 3:** Comparison of the best models, prediction accuracies, cross‐validation consistencies, and *p* values identified by GMDR analysis for HT

Best model^a^	Training balanced accuracy	Testing balanced accuracy	Cross‐validation consistency	Sign test (*p* value)
1	0.623	0.527	9/10	7 (0.356)
1,2	0.718	0.618	10/10	9 (0.019)
1,2,3	0.533	0.516	7/10	5 (0.765)
1,2,4,5	0.486	0.451	5/10	6 (0.927)
1,2,3,4,5	0.578	0.577	8/10	5 (0.386)
1,2,3,4,5,6	0.602	0.498	7/10	5 (0.459)

Abbreviation

GMDR, generalized multifactor dimensionality reduction; HT, hemorrhagic transformation.

a Numbers 1–6 represent rs3918242, rs3787268, rs1056628, rs2664517, rs17576, and rs2250889, respectively.

### 3.4 Different genotype combinations of rs3918242 and rs3787268 and HT risk

Furthermore, we investigated the associations of different genotype combination of rs3918242 and rs3787268 with risk of HT. Compared with patients carrying rs3918242TT and rs3787268AA (wild‐type genotype), the relative risk of the nine genotype combination of rs3918242 and rs3787268 for HT was assessed. The risk for HT was higher in patients harboring rs3918242CC and rs3787268GG (odds ratio [OR] =2.68, 95%CI: 1.22–5.67, *p* = 0.004), rs3918242CT and rs3787268AG (OR = 1.92, 95%CI: 1.03–4.76, *p* = 0.032), and rs3918242CC/CT and rs3787268GG (OR = 2.02, 95%CI: 1.13–5.24, *p* = 0.025), compared to those harboring rs3918242TT and rs3787268AA (Table 4). Thus, the three genotype combinations were defined as high‐risk interactive genotype. Other genotype combinations of rs3918242 and rs3787268 did not reach cutoff level of 0.05 (Table 4), and were defined as low‐risk interactive genotype.

**Table 4:** Associations between HT and genotype combinations

rs3787268	AA	GG	GG	AG	AG	GG	GG, AG	GG, AG
rs3918242	TT	CC	CC, CT	CT	CC	CT	CT	CC, CT
OR	1^a^	2.68	2.02	1.92	1.31	1.04	1.24	1.05
95% CI	—	1.22–5.67	1.13–5.24	1.03–4.76	0.92–1.96	0.97–1.73	0.74–2.28	0.82–1.87
*p* Value	—	0.004	0.025	0.032	0.226	0.328	0.588	0.675

Abbreviations

OR, odds ratio; CI, confidence interval; HT, hemorrhagic transformation.

a The low‐risk genotype for each genetic factor was used as the reference.

Incidence of HT was significantly higher in patients with high‐risk interactive genotype than those patients with low‐risk interactive genotype (22.0% [50/227] vs. 11.3% [54/478], *p* < 0.001).

#### 3.4.1 Risk factors for HT

**Figure 1:**
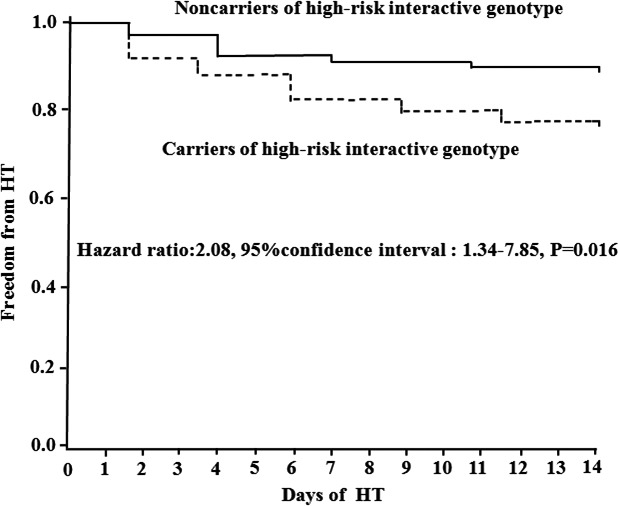
Probability of Survival Free of HT. Kaplan‐Maier analysis of cumulative freedom from HT associated with high‐risk interactive genotype (Figure 1). HT indicates hemorrhagic transformation

The risk factors for HT conferred by the combinations of rs3918242 and rs3787268 were assessed using Cox proportional hazards model. The low‐risk interactive genotype of rs3918242 and rs3787268 was assigned as zero, and the high‐risk interactive genotype was assigned as one. The other variables entered the model to adjust, including age, NIHSS score at admission, ischemic areas, cardioembolism, atrial fibrillation, and systolic blood pressure. The results showed that the high‐risk interactive genotype of rs3918242 and rs3787268 was independent predictors for the HT risk after adjustment for the covariates (HR: 2.08; 95% CI: 1.34–7.85; *p = *0.016, Table 5). Cumulative freedom for HT was lower in patients with high‐risk interactive genotype than those patients with high‐risk interactive genotype using Kaplan–Meier estimate (Figure 1).

**Table 5:** Cox regression analysis of independent predictors for HT

Factor	HR	95% CI	*p* Value
Age	0.87	0.69–1.38	0.426
NIHSS score at admission	1.62	1.08–3.76	0.032
Ischemic areas	0.82	0.91–2.56	0.268
Cardioembolism	2.31	1.48–8.35	0.006
Atrial fibrillation	0.98	0.89–2.01	0.157
Systolic blood pressure	1.01	0.91–2.23	0.224
High‐risk interactive variable	2.08	1.34–7.85	0.016

Note

HR for continuous variables means per 1‐ standard deviation increase.

Abbreviation: HT, hemorrhagic transformation; NIHSS, National Institutes of Health Stroke Scale; HR, hazard ratio; CI, confidence interval.

## 4 DISCUSSION

The possible role of genetic variants of *MMP‐9* gene in HT has not been thoroughly assessed. In this study, the results showed that the six variants of *MMP‐9* gene were not associated with the risk of HT using single‐locus analysis. GMDR analysis revealed that there was a synergistic effect of variant–variant interactions between rs3918242 and rs3787268 in *MMP‐9* gene, and the high‐risk interactive genotype was significantly associated with increased the HT risk after adjusting for the covariates.

Extensive evidence showed *MMP‐9* polymorphisms were significantly associated with carotid atherosclerosis and increased IS risk (Lin et al., 2012; Nie et al., 2014; Yuan et al., 2013). However, few studies investigated whether *MMP‐9* polymorphisms affected HT susceptibility. Zhang et al. (2015) assessed rs3918242 (C vs. T allele) polymorphisms in *MMP‐9* and found that the incidence of HT was higher in patients with CC genotype compared to those patients with CT/TT genotype, or patients carrying C‐allele compared to those carrying T allele. However, one study from Mediterranean population did not show the association between rs3918242C/T polymorphisms and HT (Montaner et al., 2003). Our current results exhibited that there was no association between the six variants of *MMP‐9* and HT risk using single‐locus analysis. There potential causes for inconsistent results may be: (a) racial differences of study populations; (b) IS and HT are complex diseases that does not follow Mendelian pattern of inheritance. Gene–environment and gene–gene interaction may play a key role in these complex diseases (Bevan et al., 2012). It is highly likely that single variant contributes to complex diseases only through their interaction with other variant (Culverhouse, Suarez, Lin, & Reich, 2002). Therefore, single‐locus linkage analysis seems unsuitable for complex genetic etiology of HT; and (c) social differences exist among different population, this may alter environmental risk to which patients are exposed.

The noteworthy observations in this study were that there was a synergistic effect of gene variant–gene variant on the risk of HT using the GMDR methods. GMDR revealed that rs3918242 and rs3787268 in *MMP‐9* had a synergistic effect to increase HT risk. The risk of HT increased by 2.08‐fold in IS patients carrying high‐risk interactive genotype of rs3918242 and rs3787268 compared with those carrying low‐risk interactive genotype, suggesting that interactions of the two variants may play an important role in genetic etiology for HT. To the best of our knowledge, current study is the first to identify that interaction of rs3918242 and rs3787268 in *MMP‐9* contribute to HT risk.

The pathophysiological mechanisms of the interactions between rs3918242 and rs3787268 in *MMP‐9* gene effect on HT susceptibility are unclear. Breakdown of BBB is one of important causes in the development of HT (Jickling et al., 2014). Previous studies have shown that MMP‐9 concentration in plasma or cerebral extracellular fluid is closely related to vascular damage, resulting from collagenase and elastase degrade extracellular matrix (Culverhouse et al., 2002). In addition, MMP‐9 plays a role in destruction and reconstruction of vascular endothelium (Hou et al., 2014), and is associated with BBB destruction and increased HT risk of acute IS (Barr et al., 2010; Castellanos et al., 2007; Jha et al., 2014; del Zoppo et al., 2012). It has been confirmed that MMP‐9 inhibitor can decrease MMP‐9 activation, neurovascular injury, BBB destruction, and incidence of HT (Lu et al., 2009, 2008). Polymorphisms of *MMP‐9* encode and regulate the transcription of MMP‐9 protein, and are associated with plasma MMP‐9 levels (Blankenberg et al., 2003). Therefore, one possible explanation for the rs3918242 and rs3787268 interaction is that the two variants participate and regulate the transcription of MMP‐9, one of the important mechanisms of HT. *MMP‐9* gene is located on the long arm of chromosome 20q13.12 in human genome, and is regulated at transcriptional level. rs3918242 variant in *MMP‐9* may inhibit protein binding, and reduce the rate of transcription and downregulate MMP‐9 expression (Lin et al., 2012). Degradation of the vascular extracellular matrix by *MMP‐9* is one important cause for vascular remodeling and angiogenesis (Hashimoto et al., 2003), and may contribute to increase the risk of intracerebral hemorrhage (Gaetani et al., 1999). Ho et al. (2015) showed that *MMP‐9* rs3787268 polymorphisms were associated with intracerebral hemorrhage (ICH), and may interact with *TIMP‐1* (tissue inhibitors of metalloproteinases) polymorphisms or alcohol to increase ICH risk. Thus, we reason that high‐risk interaction between rs3918242 and rs3787268 could provide these patients with higher MMP‐9 concentration than those patients without this particular high‐risk interaction, thereby increasing the risk of HT in acute IS.

Several limitations should be noted in this study. First, although previous study revealed *MMP‐9* polymorphisms were associated with plasma MMP‐9 levels (Blankenberg et al., 2003), plasma MMP‐9 levels were not assessed in this study. Second, because of limited samples and two‐center study, current results can not represent full spectrum of Chinese population. Thus, these findings should be confirmed in multicenter studies in future. Third, we genotyped multiple known variants in *MMP‐9* gene, some rare functional variants were not investigated in this study. Fourth, this study only investigated gene variant–gene variant interactions in six variants in *MMP‐9* gene. As previously mentioned, oxygen free radical damage, inflammation reactions, MMP‐2 and MMP‐3 may play key roles in BBB destruction and HT (Jickling et al., 2014). We did not measure the variants in oxygen free radical relevant genes, inflammation relevant genes, and *MMP‐2* and *MMP‐3* genes. Thus, a larger set of variants must be investigated to elucidate the effect of full extent of gene–gene interaction on HT susceptibility in future studies.

## 5 CONCLUSION

The six variants in *MMP‐9* were not associated with the risk of HT by single‐locus analysis. However, GMDR showed that there was a synergistic effect of gene variant–gene variant interactions between rs3918242 and rs3787268. High‐risk interactive genotype of rs3918242 and rs3787268 was significantly associated with increased risk for HT in acute IS. The GMDR analysis could provide further insight into the complex genetic pathogenesis of HT.

## CONFLICT OF INTEREST

None declared.

## DATA AVAILABILITY STATEMENT

The data that support the findings of this study are available on request from the corresponding author. The data are not publicly available due to privacy or ethical restrictions.
